# Design, construction, and calibration of a weighable lysimeter for measuring water requirements of field crops for data-scarce areas of Ethiopia

**DOI:** 10.1016/j.heliyon.2024.e36968

**Published:** 2024-08-27

**Authors:** Nigusie Kebede, Mekonen Ayana, Boja Mekonnen, Habtamu Beri

**Affiliations:** a Department of Water Resources Engineering, Adama Science and Technology University Adama, Ethiopia; b School of Mechanical, Chemical and Materials Engineering, Adama Science and Technology University Adama, Ethiopia

**Keywords:** Lysimeter, Weighing, Portable, Water use, Shallow-rooted crops

## Abstract

A simple weighable lysimeter was designed, constructed, and calibrated for measuring the water requirements and crop coefficients of shallow-rooted crops. It has a portable weighing mechanism to quantify changes in soil moisture content. The weighing mechanism consists of a horizontal steel bar, a hydraulic car jack, both ends hooked vertical steel bars, a stand, and a digital weighing balance. As the lysimeter's inner tank, a plastic drum with a 55 cm diameter that yields 0.24 m^2^ of internal area was used. A performance test was conducted, and a rating curve was developed at the beginning of the growing seasons in 2023 and 2024 to evaluate the sensitivity of the weighing mechanism in determining crop water use. Linearity, repeatability, and measurement uncertainty tests were conducted. The linearity error was ±0.04 kg and ±0.03 kg for the years 2023 and 2024, respectively, which is within the allowable limit. The weighing system repeats the measurement perfectly with no error (repeatability error was zero). The combined uncertainty of the measurements was 0.023 kg, representing the cumulative effect of individual errors. The lysimeter weighing system is capable of detecting variations in mass as small as 0.2 mm of moisture content. Therefore, the developed lysimeter can be used to determine water requirements and crop coefficients of shallow-rooted crops for the design and management of irrigation in data-scarce areas of Ethiopia.

## 1 Introduction

Lysimeters are soil-filled tanks or containers where plants are grown and applied to monitor how much water is consumed by a vegetated surface in evapotranspiration. It is also used in determining the chemical mobility in the soil profile in water quality investigations [1]. Lysimeters used in evapotranspiration research are often categorized by gravity or vacuum drainage, weighing or non-weighing, and monolithic or rebuilt soil profiles [2]. Different lysimeter types have been reported [1,[3], [4], [5], [6], [7], [8], [9], [10]]. Basically, common lysimeters are of two kinds: non-weighing and weighing [6].

Weighing lysimeters are standard tools [11], the most accurate technique to measure crop evapotranspiration [12], and used to verify or calibrate weather-based reference evapotranspiration estimation models [13]. These are true, provided that they are appropriately designed, built, installed, and operated [14,15]. The lysimeter weight difference before and after a certain period (Δt) yields data on the average change in soil water content (ΔS) [9]. This gives the crop's average evapotranspiration rate, together with other elements of the soil water balance (irrigation, precipitation, and drainage).

Weighing lysimeters are classified as either continuous or intermittent (weighable lysimeters), depending on how long it takes between two successive weight measurements [16]. The former are not commonly utilized because of their high installation costs and need for specialized workers, even if they are accurate and precise [17].

With this kind of lysimeter, successive measurements are collected in a 1-min time interval, and both the lysimeter and the weighing mechanism are permanently installed [18]. In weighable lysimeters, successive measurements are gathered over a day or longer time interval, and the weighing facility moves to the lysimeter each time to take readings since it is movable.

Lysimeters’ main objective in evapotranspiration determination is to create a controlled environment enabling the monitoring of water entering and leaving the system. This needs matching the soil-plant system within the lysimeter with respect to soil water content [19], plant density, soil or nutrient availability [4], and other factors with the surrounding environment. However, it is challenging to match the water and soil conditions both inside and outside the lysimeter. For instance, more moisture is available at the bottom of the soil profile of a lysimeter relative to the surrounding area of the same depth, unless an efficient drainage system drains the surplus water [20]. To minimize this problem, great concern is required at every stage of the design, building, installation, and field management of the lysimeters.

Lack of site and crop-specific data is hindering efficient and effective design and management of irrigation in Ethiopia. Lysimeters are the old and still useful tools to directly measure water requirements and crop coefficients, which are important for efficient irrigation management. In Ethiopia, a limited number of lysimeters are available (at Melkasa, Werer, and Bishoftu Agricultural Research Centers). In all sites, the type of lysimeter is a non-weighing type; soil moisture change is monitored using a neutron probe, which is expensive. From this perspective, this study was aimed to design, construct, and calibrate a simple weighable lysimeter to measure water requirements and crop coefficients of irrigated shallow-rooted crops.

## 2 Material and methods

### 2.1 Design and construction of lysimeter

In designing the weighable lysimeter for measuring crop evapotranspiration, the following three points were considered as a design criterion, which are very important. First, the lysimeters needed to be big enough to replicate field circumstances; second, they needed to be small enough to avoid requiring pricey lifting and weighing equipment, as stated by Martin et al. [1]; and third, the weighing scale needed to be readily available on the local market. Based on the aforementioned requirements, a system for crops with shallow roots was designed (onion was taken as a test crop).

In November 2022, the development of the lysimeters started with excavation work for two lysimeters, a drainage system, and an access chamber. It was completed by constructing and installing the lysimeters, drainage system, lifting, and weighing mechanism for the growing seasons of 2023 and 2024. In all the two years, the same lifting and weighing mechanism was used. In these two consecutive growing seasons, the lysimeters were utilized to study onion water requirements.

**Fig. 1:**
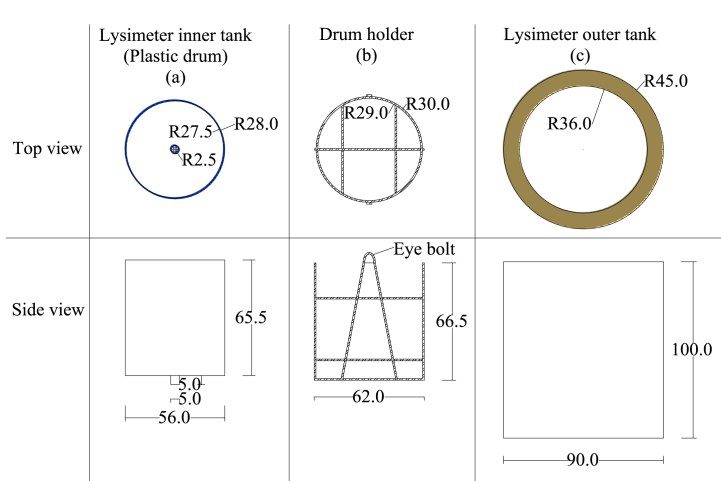
Top and side views of the design drawings for the inner tank (a), drum holder (b), and outer tank (c); all the dimensions are in centimeter.

As a component, each lysimeter contained a circular inner and outer tank. As the inner tank, a plastic drum with a 55 cm internal diameter, 0.5 cm thickness, and 65 cm depth, which yields 0.24 m^2^ of internal surface area, was used (Fig. 1a). This plastic drum was placed into the drum holder built from a steel bar that had two eye bolts placed face-to-face with each other to support the plastic drum during lift. This component was built from a steel bar of 10 mm in diameter (Fig. 1b). A concrete pipe was used as an outer tank to avoid contact between the inner tank and the surrounding soil. It was sized to a 5 cm gap all around the drum holder to avoid wall contact as well as to allow up and downward movement of the inner tank during weighing (Fig. 1c). To allow the drainage of any water that could fall between the inner and outer tanks into the ground, the bottom of the concrete pipe was left open.

**Fig. 2:**
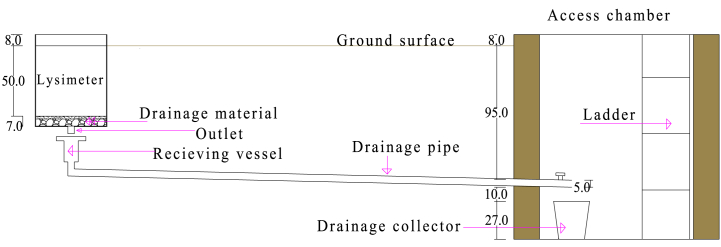
A design drawing of the drainage system showing the drainage facility within the lysimeter, the drainage pipe, and the drained water collector at the access chamber.

The gravity drain type of drainage system was designed for the lysimeters to maintain the same soil moisture profiles within and out of the lysimeter (Fig. 2). To facilitate drainage at the bottom of the inner tank, 5 cm thickness of gravel (pebbles having 1–2 cm diameter) and 2 cm thickness of sand were provided. Moreover, to remove the excess water, a 50-mm-diameter hole (outlet) at the base of the inner tank was provided, which was centered and mounted with a 2 inch shower drain having a 50 mm depth. Further, the drainage system had two components: the drainage pipe and the drained water collector.

The drainage pipe used was a pipe system that was used to transport drained water from the outlet of the inner tank to the drained water collector, located at the access chamber. It was composed of a receiving vessel and 50-mm-diameter PVC aligned horizontally to the access chamber with a smooth gradient. The receiving vessel comprises a funnel made of an iron sheet that had a 23 cm diameter and 110 mm diameter PVC, which was reduced to 50 mm diameter using a reducer. This receiving vessel had a 28 cm height and was buried vertically at the center of the outer tank, 7 cm below the bottom of the inner tank. The drained water was collected at the drain collector, which had a 10-L capacity, dimensions of 27 cm in height, and a 28 cm top diameter. The drained water between two irrigation intervals was measured at the access chamber, which had a 1.2 m width, 1.5 m length, and 1.4 m depth.

The drainage system has three components: the drainage system in the lysimeter, the drainage pipe, and the drained water collector. The drainage pipe and the drained water collector work together to transport and collect the drained water from the lysimeter, respectively. The drainage system within the lysimeter is crucial for maintaining consistent soil moisture conditions both inside and outside the lysimeter. This is achieved through.

• Matching the characteristics of the soil inside the lysimeter with the surrounding. This was achieved through backfilling the soil in the natural order where it was removed and careful compacting during backfilling. This creates similarities in water retention and movement properties.
• Providing a drainage material and outlet at the bottom to remove the excess water drained to prevent excess moisture within the lysimeter.

### 2.2 Lifting and weighing system

The lifting and weighing system's design requirements were to: 1) make it simple to transfer among lysimeters; 2) make it simple to remove from the site so as not to obstruct field activities; and 3) have enough strength to support the loads, as adopted from Martin et al. [1].

**Fig. 3:**
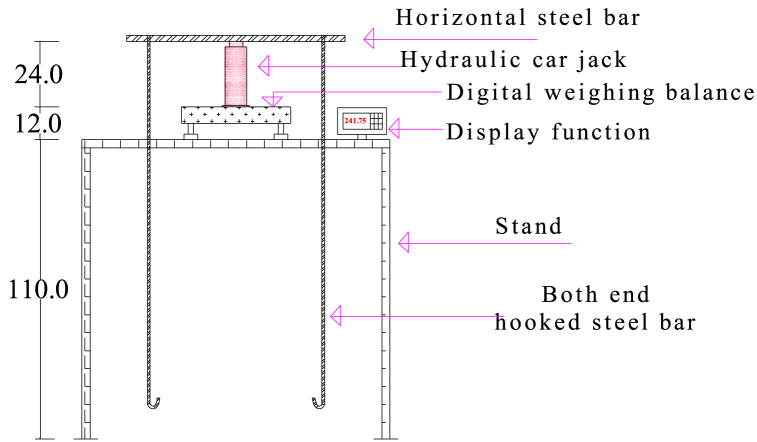
Side view design drawing of a lifting and weighing system, which is moved to the lysimeter position and placed at the ground surface to lift the lysimeter for measurement.

The lifting system was constructed from four different components (Fig. 3). The first component is a horizontal steel bar, which is placed at the top of the hydraulic car jack and used to suspend the lysimeter during the lift for weighing. The steel bar having the capacity to hold the load caused by the mass suspended on it without bending was selected. For this case, a steel bar with a diameter of 24 mm and a length of 80 cm was used. The second component is the hydraulic car jack, which is used to lift all the loads suspended on the steel bar by bushing up. So, a hydraulic car jack with a lifting capacity of 10 tons was selected in respect of stability during lift, even if the required lifting capacity was 262 kg. The third component is the stand, a table-like structure measuring 110 cm above the ground used to place the weighing platform. The fourth component is two steel bars, 12 mm in diameter and 130 cm in length, hooked on both ends with removable links that were used to connect the eye bolts on the drum holder and the horizontal steel bar for lifting the lysimeter. This bar could withstand a tension load caused by the total mass within the lysimeter.

The weighing system used was a digital scale weighing platform floor scale, Model No. TESA33001, with a 300 kg and 1.5 kg maximum and minimum capacity, respectively, with a division of 50 g. It has a display function and measures the weight directly. This weighing system was selected based on its suitability for the designed system, availability in the market, and relatively low cost (Fig. 3).

### 2.3 Lysimeter installation

**Fig. 4:**
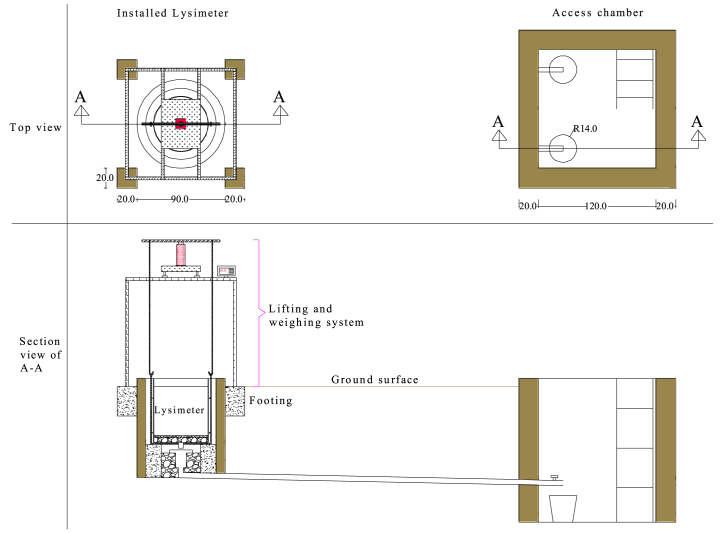
A design drawing of the installed lysimeter at Melkasa Agricultural Research Center; showing the system components: the lysimeter, drainage system, lifting, and weighing system.

The lysimeters were installed at Melkasa Agricultural Research Center (MARC), which is found in the Southeast Shoa Zone of Oromia regional state of Ethiopia. The lysimeters were installed at a non-weighing type lysimeter experiment site at the research center. The assembled and installed lysimeter drawing with all its components is shown in Fig. 4.

At each lysimeter location, a circular pit that is slightly deeper and larger than the outer tank was dug, and at the access chamber location, a rectangular pit considering the working space was dug. For the drainage pipes, a canal that connects the center of each lysimeter location with the access chamber, having a depth less than the height of the drainage collector from the depth of the access chamber, was dug. During excavation of the circular pit and canal, the soil was removed in a 10 cm layer and placed in a separate pile for backfill.

The drainage pipes were then installed carefully. The bottom of the lysimeter pit was leveled, lean concrete was placed, leaving a 40 cm diameter circular area at the center, and the outer tank was lowered into the pits. The opening surrounding the outer tank was backfilled with soil, and 30 cm thick concrete was placed inside the outer tank, leaving a 40 cm diameter circular area at the center. Then, the annular area between the receiving vessel and this 40 cm diameter circular area was filled with gravel to facilitate the drainage of water that might enter between the inner and outer tanks.

The drum holder was lowered into the outer tank, and the inner tank was placed inside the drum holder by aligning the outlet on the inner tank with the receiving vessel. In each inner tank, a drainage material, a 5 cm layer of gravel (pebbles having 1–2 cm diameter), and 2 cm of sand were provided. The rest of the inner tank, leaving the rim and the canal for the drainage pipe, were filled back using the initial soil in the opposite order that it was removed. Finally, four footings at the outer side of the corners of a square, which inscribe the outer tank, had a 20 cm by 20 cm dimension at surface level for the stand using concrete, and the access chamber using hollow blocks were constructed. The drained water collectors were placed in the access chamber at the outlet position of the drainage pipes.

### 2.4 Operational principle

**Fig. 5:**
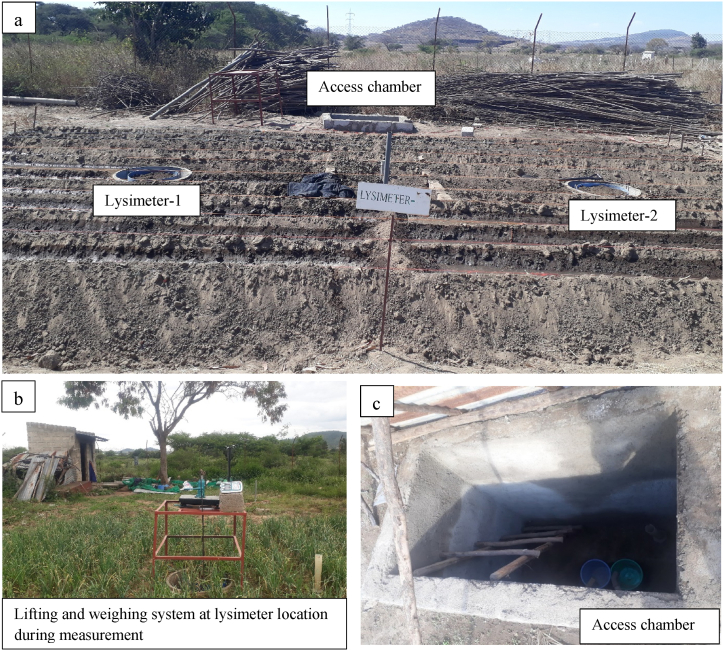
Lysimeters with access chamber (a), lifting and weighing systems (b), and inside of the access chamber (c).

The stand was positioned on the footings, and the weighing platform was placed on the stand with the hydraulic car jack on top of it. On the top of the hydraulic car jack, the horizontal steel bar was placed, and this bar was connected to the eyebolts on the drum holder using vertical removable steel bars hooked at both ends. A section view of the installed lysimeter in Fig. 4 and the real-life image in Fig. 5 show the arrangement.

When the hydraulic car jack operated (raised), the horizontal steel bar was pushed up, and since this bar was connected to the eyebolts on the drum holder, the lysimeter started to be lifted. As the lysimeter gets lifted, the weight is displayed on the display function. After taking the reading, the lysimeter is then moved down until it reaches the floor by reducing the height of the hydraulic car jack. Then, the weighing and lifting systems were moved to the other lysimeter.

### 2.5 Calibration and performance testing

A performance test was undertaken to ensure the reliability of the weighing system and adherence to the manufacturer's specifications. A weighing system evaluation was conducted at the start of the 2023 and 2024 growing seasons.

#### 2.5.1 Calibration

Calibration is the process of comparing a mass standard's weight reading with its actual value [21]. Daniel et al. [22] described that calibration of the lysimeter is performed by loading and unloading known weights (weights that are standardized by weighing on an electronic scale with high resolution). However, in this case, calibration was done by loading known weights within the calibration range; since the weighing system is intermittent, there is no need for a hysteresis check.

The calibration range considered was the mass between 70 % of total available water depleted and field capacity (10.5 kg). The calibration range, known as the operating range, is the portion of the instrument's measurement range across which it is calibrated to provide accurate and reliable measurements. In this study, the operating range (commonly used measurement range) considered was the mass of readily available soil water (RAW) in the root zone. This readily available soil water is the average portion (p) of total available soil water (TAW) that can be used up before moisture stress (a decrease in ET) occurs. The value of p varies among crops, and it goes to 0.7, which implies readily available water is 70 % of TAW [23]. Even if the test crop was an onion with a p value of 0.3, the study considered crops with a maximum p value of (0.7) to widen the operating range for different crops.

So, this weight, i.e., the mass between 70 % of total available water depleted and field capacity, was divided into six different known weights (considering the division of the weighing scale) to produce calibration points. Known weights were prepared from packed bricks in plastic bags using a scale with a 0.01 g resolution. These masses were applied continuously in increasing steps on the constant load of the lysimeter, 236.6 kg, taken as the standard weight to be weighted on the lysimeter.

At each calibration point, pairs of data (the true value and the lysimeter reading of the standard mass) were collected. These data were subjected to a linear regression analysis as explained by Sanches et al. [15], and then a calibration curve was produced. This curve (regression line or best-fit equation), often called the transfer function, shows how the measurement values are related to a recognized reference.

#### 2.5.2 Performance test

The performance test of the weighable lysimeter was analyzed by evaluating linearity, repeatability, and measurement uncertainty parameters adopted from Martin et al. [1].

Linearity is the characteristic that measures the accuracy of the instrumental intermediate readings across the instrument's weighing range [21]. The relationship between the true value of the load and the reading of the scale is called its characteristic curve; ideally, it is a straight line. As described by Morse and Baer [21], the deviation of the characteristic curve from this straight line going through the weighing range is called non-linearity.

The data collected from the calibration procedure was used to analyze the system's linearity. The deviation or difference between each measured value and the best-fit equation was evaluated for each measured value. The linearity error, or maximum deviation, is compared to the manufacturer's specifications [1].

Repeatability refers to the capability of a device to yield the same result when an identical load is applied several times in sequence to the weighing system using the same procedure. As described by Walendziuk and Idzkowski [24], the test is undertaken with a test load of about 50 % of the calibration range, and one repeatability test consists of 10 repeated measurements of the same mass. It is expressed as a standard deviation [21,24] and calculated using Equation (1).

(1) $$s\left(m\right)=\sqrt{\sum_{j=1}^{N}\frac{{\left(m_{j}-\bar{m}\right)}^{2}}{N-1}\;}$$

Where, $s\left(m\right)=standard\;devation;\;m_{j}=value\;j\;of\;measured\;mass,\bar{m}=mean\;mass\;and\;N=number\;of\;samples$.

The maximum standard deviation is the repeatability error; it is compared with the manufacturer's specifications [25].

For this study, three test loads from the calibration range were taken; each test load was measured 10 times, and the standard deviations were computed using Equation (1).

The tests in this study were undertaken in a relatively closed environment using a standard load instead of using the lysimeter in the field to reduce environmental effects such as the wind effect.

Measurement uncertainty describes the doubt that exists around the outcome of any measurement. The steps taken to compute the uncertainty of a measurement were identifying the sources of uncertainty, quantifying the uncertainties, and combining the uncertainties to provide an overall figure.

The standard uncertainty of linearity and repeatability error for linearity and repeatability measurements were computed using Equations (2), (3)), respectively.

(2) $$u_{Le}=\frac{a}{\sqrt{3}}$$

Where, $u_{Le}=standard\;uncertainity\;of\;linearity\;error\;and\;a=half\;range\;of\;the\;error$.

(3) $$u_{Re}=\frac{s}{\sqrt{n}}$$

Where, $u_{Re}=standard\;uncertainity\;of\;repeatability\;error\;and,s=standard\;deviation\;and\;n=number\;of\;measurments$.

The combined standard uncertainty of the measurements was computed using Equation (4).

(4) $$u_{c}=\sqrt{u_{Le}^{2}+u_{Re}^{2}}$$

 Where, $u_{c}=combined\;standard\;uncertainity,u_{Le}=standard\;uncertainity\;of\;linearity\;error\;and,u_{Re}=standard\;uncertainity\;of\;repeatability\;error$.

### 2.6 Validation

To confirm the validity of the developed lysimeter, the stage-based crop coefficient of the developed lysimeter for the test crop was compared with the FAO-56 value as described by M. Soler-Méndez [10]. The crop coefficient of the test crop for the developed lysimeter was determined as a ratio of crop evapotranspiration (measured using the lysimeter) and reference crop evapotranspiration (computed using CROPWAT version 8.0).

## 3 Results and discussions

### 3.1 Calibration

**Fig. 6:**
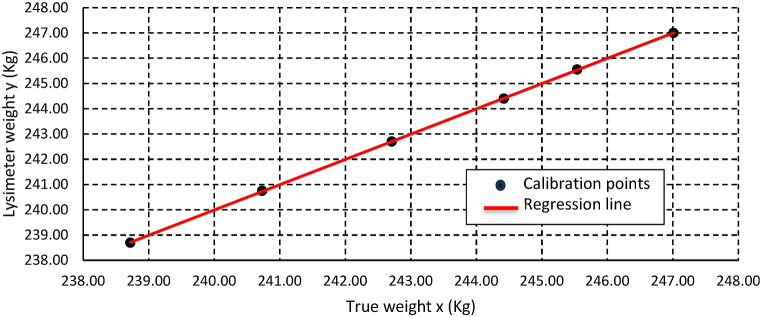
Calibration curve for the year 2023.

The calibration curve produced for the weighing lysimeter follows the calibration points perfectly. The linear regression coefficient for both years and the plot of lysimeter weight (measured weight) versus true weight for the year 2023 are presented in Table 1 and Fig. 6, respectively.

**Table 1:** Linear regression coefficient for the weighing system calibration curve.

Year	Test range (kg)	Equation coefficient^a^		R^2^
		b	c	
**2023**	10.41	1.0001	−0.0337	0.999
**2024**	10.53	1.0010	−0.2380	0.999

a y = bx + c where y - dependent variable, presents lysimeter weight (kg) and x - independent variable, presents the true weight (kg).

### 3.2 Linearity test

**Fig. 7:**
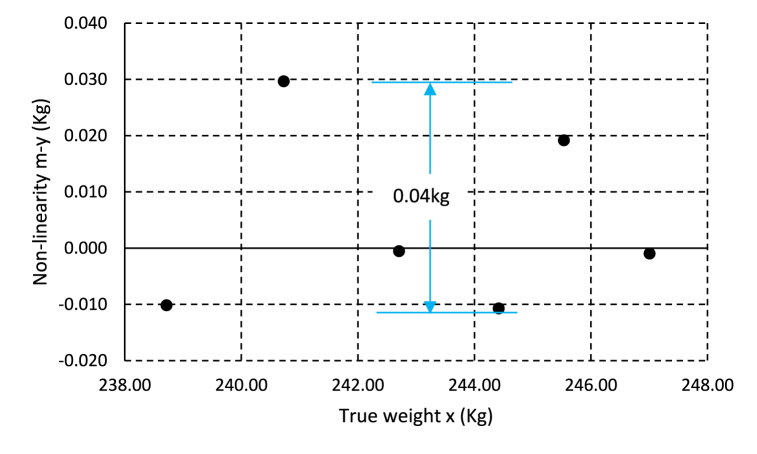
Residuals from the weighing lysimeter calibration test for the year 2023.

The data collected from the calibration procedure was used to analyze the system's linearity. The deviation or difference between each measured value (lysimeter reading) and the best-fit equation (m-y) was evaluated for each measured value. The linearity error, or maximum deviation, is compared to the manufacturer's specifications. The linearity error was ±0.04 kg and ±0.03 kg for the tested range of 10.41 kg and 10.53 kg for the years 2023 and 2024, respectively. The linearity error for the test year 2023 was presented in Fig. 7. Per the standards provided by the manufacturer, the non-linearity ought to be ±0.05 kg. Thus, the system's linearity meets the manufacturer's specifications.

### 3.3 Repeatability test

**Fig. 8:**
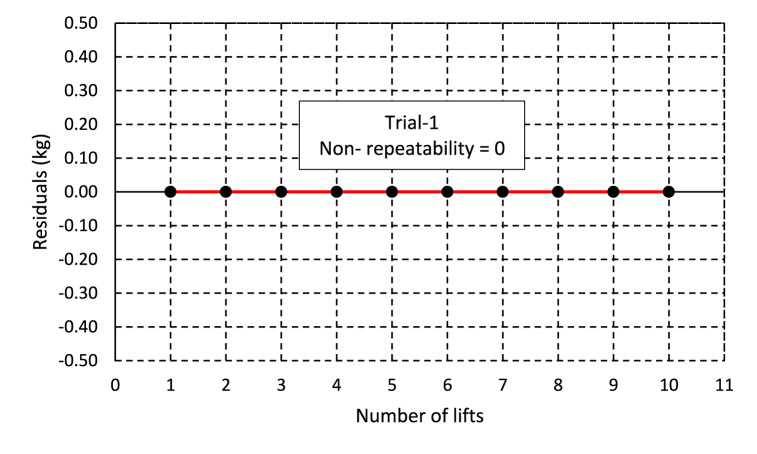
Repeatability error of the weighing system for trial-1 of 10 lifts.

Data for the repeatability test was collected by loading three test loads as a trial with different weights on a standard load of 236.6 kg and 237.05 kg for the years 2023 and 2024, respectively. The repeatability error was measured using the residual average spread, or the average spread of the difference among the average and measured weight for each lift, described as a standard deviation. For all three trial loads in both years, the analysis showed a zero-standard deviation, which means the weighing system repeats the measurement perfectly with no error. The reason for the claim of zero repeatability error is due to the characteristic of the weighing system. The weighing system used has a division of 50 g; this means it cannot detect small changes within this range. This made the measurement repeatable without error. Fig. 8 displays the trial's residual in relation to the number of lifts.

### 3.4 Measurement uncertainty

After the errors for linearity and repeatability measurements were quantified, the standard uncertainty of measurement for each error and for the combined effect was computed. The result showed that for the linearity and repeatability error of ±0.04 kg and 0 (zero), the standard uncertainty of measurements was found to be 0.023 kg and 0 (zero), respectively. The combined standard uncertainty of the measurements was 0.023 kg. This analysis shows repeatability error makes no contribution to the total uncertainty, whereas linearity error was the main cause.

### 3.5 Resolution

The lysimeter weighing system is capable of detecting variations in mass as small as 50 g, or 0.2 mm of water head.

### 3.6 Validation

Stage-based crop coefficients of the developed lysimeter for the test crop (Onion) were found to be 0.68, 1.03, and 0.86 for the initial, mid-season, and late-season stages, respectively. In comparison, the FAO-56 [23] suggested crop coefficient values of 0.7, 1.05, and 0.75 for these stages. The close similarity between these values confirms that the measurements from the developed lysimeter are acceptable and validates its design for the intended function.

### 3.7 Limitations and future work

The developed lysimeter was designed for shallow-rooted crops, but to be applicable for a wider range of crops, the designed lysimeter has two limitations. First, the lysimeter's depth is small, with an overall depth of 65 cm and a net soil depth of 50 cm. To accommodate deep-rooted crops, the -soil profile in the lysimeter must be deeper to match the root depth of the crop. Increasing the depth while maintaining the same surface area results in a greater volume and, consequently, a higher mass, which necessitates a larger lifting and weighing system. Second, the capacity of the current lifting and weighing system is limited, with a maximum weighing capacity of 300 kg. This limitation in the stand height of the lifting system and the weighing system's capacity restricts the use of the lysimeter for deep-rooted crops.

## 4 Conclusion

The weighable lysimeter detailed in this study was utilized in the 2023 and 2024 growing seasons at Melkasa Agricultural Research Center. The system did not encounter significant operational problems, and the developed weighing, lifting, and drainage system was successful. Data were gathered on the water use of onions.

Because of the weighing system's portability, issues with spatial variability in field research can be resolved by distributing numerous lysimeters randomly over an experimental plot. The weighing system generated acceptably accurate data that could be used for determining crop water requirements and crop coefficients for several crops grown in Ethiopia.

## Data availability statement

Data will be made available on request.

## CRediT authorship contribution statement

**Nigusie Kebede:** Writing – original draft, Methodology, Investigation, Formal analysis, Conceptualization. **Mekonen Ayana:** Writing – review & editing, Supervision, Resources, Methodology, Conceptualization. **Boja Mekonnen:** Writing – review & editing, Supervision, Methodology. **Habtamu Beri:** Writing – review & editing, Supervision, Methodology.

## Declaration of competing interest

The authors declare that they have no known competing financial interests or personal relationships that could have appeared to influence the work reported in this paper.

## References

[1] Martin E.C., De Oliveira A.S., Folta A.D., Pegelow E.J., Slack D.C. (2001). Development and testing of a small weighable lysimeter system to assess water use by shallow–rooted crops. Transactions of the ASAE.

[2] Kohnke F.R., Dreibelbis H. (1940).

[3] Gifford H.H., Whitehead D., Thomas R.S., Jackson D.S. (1982). Design of a new weighing lysimeter for measuring water use by individual trees. New Zeal. J. For. Sci..

[4] Khan B.R., Mainuddin M., Molla M.N. (1993). Design, construction and testing of a lysimeter for a study of evapotranspiration of different crops. Agric. Water Manag..

[5] Payero J.O., Irmak S. (2008). Construction, installation, and performance of two repacked weighing lysimeters. Irrig. Sci..

[6] Parisi S., Mariani L., Cola G., Maggiore T. (2009). Mini-lysimeters evapotranspiration measurements on suburban environment. Ital. J. Agrometeorol..

[7] Fisher D.K. (2012). Simple weighing lysimeters for measuring evapotranspiration and developing crop coefficients. Int. J. Agric. Biol. Eng..

[8] Lorite I.J., Santos C., Testi L., Fereres E. (2012). Design and construction of a large weighing lysimeter in an almond orchard. Spanish J. Agric. Res..

[9] Nicolás-Cuevas J.A., Parras-Burgos D., Soler-Méndez M., Ruiz-Canales A., Molina-Martínez J.M. (2020). Removable weighing lysimeter for use in horticultural crops. Appl. Sci..

[10] Soler-Méndez M., Parras-Burgos D., Mas-Espinosa E., Ruíz-Canales A., Intrigliolo D.S., Molina-Martínez J.M. (2021). Standardization of the dimensions of a portable weighing lysimeter designed to be applied to vegetable crops in mediterranean climates. Sustain. Times.

[11] Ünlü M., Kanber R., Kapur B. (2010). Comparison of soybean evapotranspirations measured by weighing lysimeter and Bowen ratio-energy balance methods. Afr. J. Biotechnol..

[12] Johnson R.S., Williams L.E., Ayars J.E., Trout T.J. (2005). Weighing lysimeters aid study of water relations in tree and vine crops. Calif. Agric..

[13] Fenner W., Dallacort R., Junior C.A.F., Freitas P.S.L.D., Queiroz T.M.D., Santi A. (2019). Development, calibration and validation of weighing lysimeters for measurement of evapotranspiration of crops. Rev. Bras. Eng. Agric. e Ambient..

[14] Bryla D.R., Trout T.J., Ayars J.E. (2010). Weighing lysimeters for developing crop coefficients and efficient irrigation practices for vegetable crops. Hortscience.

[15] Sanches A.C., Souza D.P.d., Mendonça F.C., Maffei R.G. (2017). Construction and calibration of weighing lysimeters with an automated drainage system. Rev. Bras. Eng. Agric. e Ambient..

[16] Howell T.A., Schneider A.D., Jensen M.E. (1991). History of lysimeter design and use for evapotranspiration measurements. Lysimeters Evapotranspiration Environ. Meas..

[17] Howell T.A., McCormick R.L., Phene C.J. (1985). Design and installation of large weighing lysimeters. Trans. Am. Soc. Agric. Eng..

[18] Zupanc V., Nolz R., Cepuder P., Bračič-Železnik B., Pintar M. (2012). Determination of water balance components with high precision weighing lysimeter in Kleče. Acta Agric. Slov..

[19] Shenkut A., Tesfaye K., Abegaz F. (2013). Determination of water requirement and crop coefficient for sorghum (sorghum bicolor L.) at melkassa, Ethiopia. Sci. Technol. Arts Res. J..

[20] Tanner C.B. (2015). Measurement of evapotranspiration. Irrig. Agric. Lands.

[21] Morse D., Baer D.M. (2004). Laboratory balances: how they work, checking their accuracy. Lab. Med..

[22] Daniel C. (2013). Design , installation and calibration of a weighing lysimeter for crop evapotranspiration studies para estudos de evapotranspiração de culturas agrícolas. Water Resources and Irrigation Management.

[23] Allen R.G., Pereira L.S., Raes D., Smith M. (1998).

[24] Walendziuk W., Idzkowski A. (2017). Postupak baždarenja i analiza nesigurnosti elektronske vage na temelju strujnog kruga istosmjerne i izmjenične struje. Teh. Vjesn..

[25] NATA (2018).

